# Idiopathic Eruptive Cherry Angiomatosis: A Case Series of Three Healthy Women

**DOI:** 10.7759/cureus.90893

**Published:** 2025-08-24

**Authors:** Adriana Campo, Travis Frantz, Monica L Borza, David S Kirwin, Willis H Lyford

**Affiliations:** 1 Department of Dermatology, Naval Medical Center San Diego, San Diego, USA

**Keywords:** angioma, dermatology, eruptive cherry angiomatosis, female patients, patient counseling, red bumps, skin growth

## Abstract

Eruptive cherry angiomatosis (eCA) is a rare clinical diagnosis. The underlying pathophysiology is unknown, but eCA has been associated with medications, lymphoproliferative disease, graft versus host disease, immunosuppression, and human herpesvirus-8. To our knowledge, there have not been previous reports of eCA in healthy female patients. The goal of this case report is to describe the clinical characteristics and present a novel case series of three otherwise healthy adult women and attempt to characterize common features, comparing patient demographics, any associated comorbidities, clinical presentation, and treatment. Three female patients (aged 52, 42, and 38 years) from Southern California were diagnosed with eCA. All three patients were experiencing hormonal fluctuations at the time of onset, including perimenopause and pregnancy. Despite stabilization of their hormonal conditions, their lesions have persisted. Patient A has had her cherry angiomas treated several times with a 595 nm pulse dye laser; however, her lesions have continued to recur. The other two patients have not sought treatment. All patients are in good health without any significant comorbidities.

## Introduction

Eruptive cherry angiomatosis (eCA) is a rarely reported condition characterized by an eruption of hundreds to thousands of benign, asymptomatic cherry angiomas. It has been associated with medications and systemic illness [1]. This eruption can be cosmetically distressing, and patients may initially seek dermatologic care for cosmetic management or to determine the underlying cause. It is important for clinicians to be aware of this condition, potential underlying etiologies, response to treatment, as well as more recent literature that suggests an association between malignant melanoma and eCA [2-3]. eCA may be underreported as these lesions are asymptomatic, and there is no pathognomonic number of lesions or time in which they develop to make the clinical diagnosis. More research is needed to understand the pathophysiology and associated conditions of this disorder. To help increase awareness, we present the cases of three healthy female patients who developed eCA while undergoing hormonal changes.

## Case presentation

Patient A

A 52-year-old woman with an unremarkable history presented to her primary care provider in September 2022 with increased bruising for four weeks and irregular periods for four months in the setting of a sharp increase in cherry angiomas since March 2021. Workup was notable only for microscopic hematuria, and the patient was referred to a community dermatologist for treatment of her cherry angiomas, which were cosmetically distressing. 

Microscopic hematuria persisted into April 2023, which prompted urology referral and a CT abdomen/pelvis, which revealed two hepatic hemangiomas (5.7 cm, 6.6 cm), soft tissue fullness in the vaginal apex/cervix, and a small, nonobstructing left intrarenal calculus. Urology workup, including cystoscopy, was reassuring, so she was recommended annual urinalysis until hematuria resolves, versus repeat urology consult if persistent over the next three to five years. Gastroenterology consult deemed her hepatic angiomas as asymptomatic, but should this change, surgical treatment was decided to be considered vs embolization. In addition, the patient completed age-appropriate cancer screenings. Her pap smear and mammogram were negative. Perimenopause was suspected for the change in her menstrual cycle. However, a pelvic ultrasound was ordered to further characterize the CT findings, ultimately negative for malignancy. 

**Figure 1:**
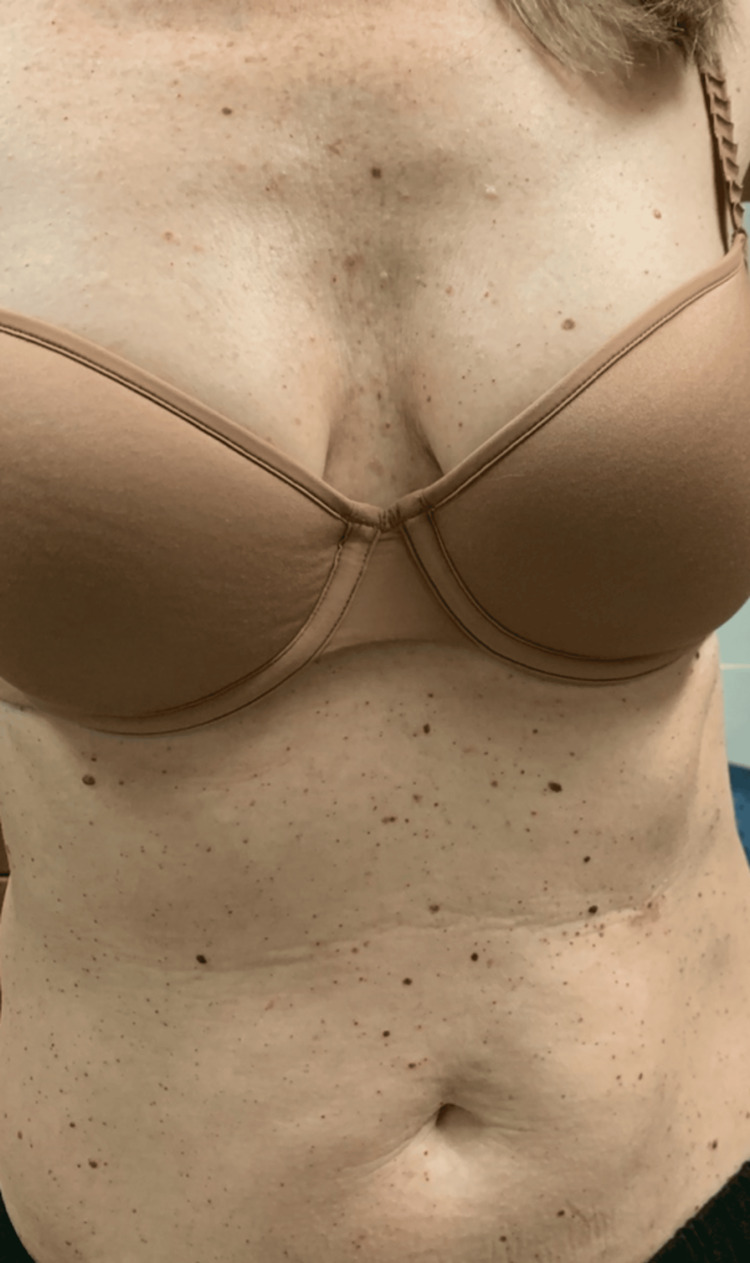
Numerous cherry hemangiomas on anterior trunk of Patient A

The patient presented to our dermatology clinic for a second opinion on her skin lesions (Figure 1) in December 2023. She had been seen by a community dermatologist who had no concern for underlying disease and had treated her cherry angiomas with PDL twice. Unfortunately, despite treatment, the cherry angiomas recurred at the treatment site in approximately the same number and density as prior to treatment. A biopsy was performed, which confirmed that the lesions were hemangiomas. An exhaustive review of her medical record was conducted without significant findings. The patient was treated with PDL using purpuric settings in our clinic; unfortunately, the lesions recurred in the treated area.

Patient B

**Figure 2:**
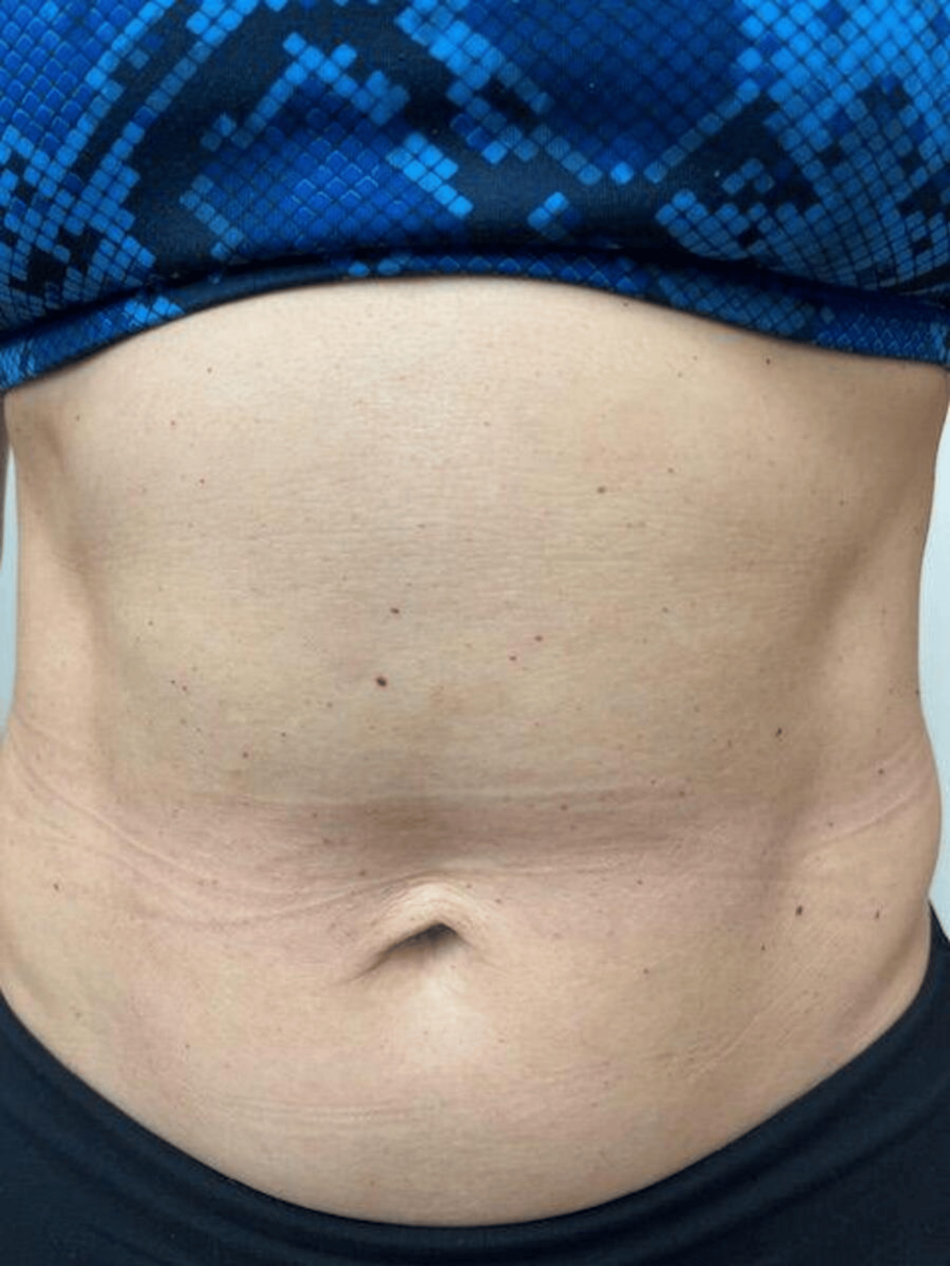
Initial anterior trunk presentation, consistent with cherry hemangioma, in Patient B

A 38-year-old G1P1 female patient presented to our dermatology clinic for evaluation of numerous asymptomatic small red raised lesions on her trunk, which suddenly appeared after the delivery of her first child three years previously (Figure 2). Her pregnancy history was notable for advanced maternal age, stage one hypertension, and noninflammatory pericardial effusion. She also suffered from postpartum thyroiditis. The patient's primary care manager started laboratory tests for symptoms of hypothyroidism vs sequelae of post-partum state in June 2023 (delivery date July 2022), which revealed thyroid-stimulating hormone (TSH), but elevated thyroid antibodies. At presentation to our clinic in July 2024, TSH was normal; anti-thyroid peroxidase and thyroglobulin antibodies were elevated but trending downward. 

**Figure 3:**
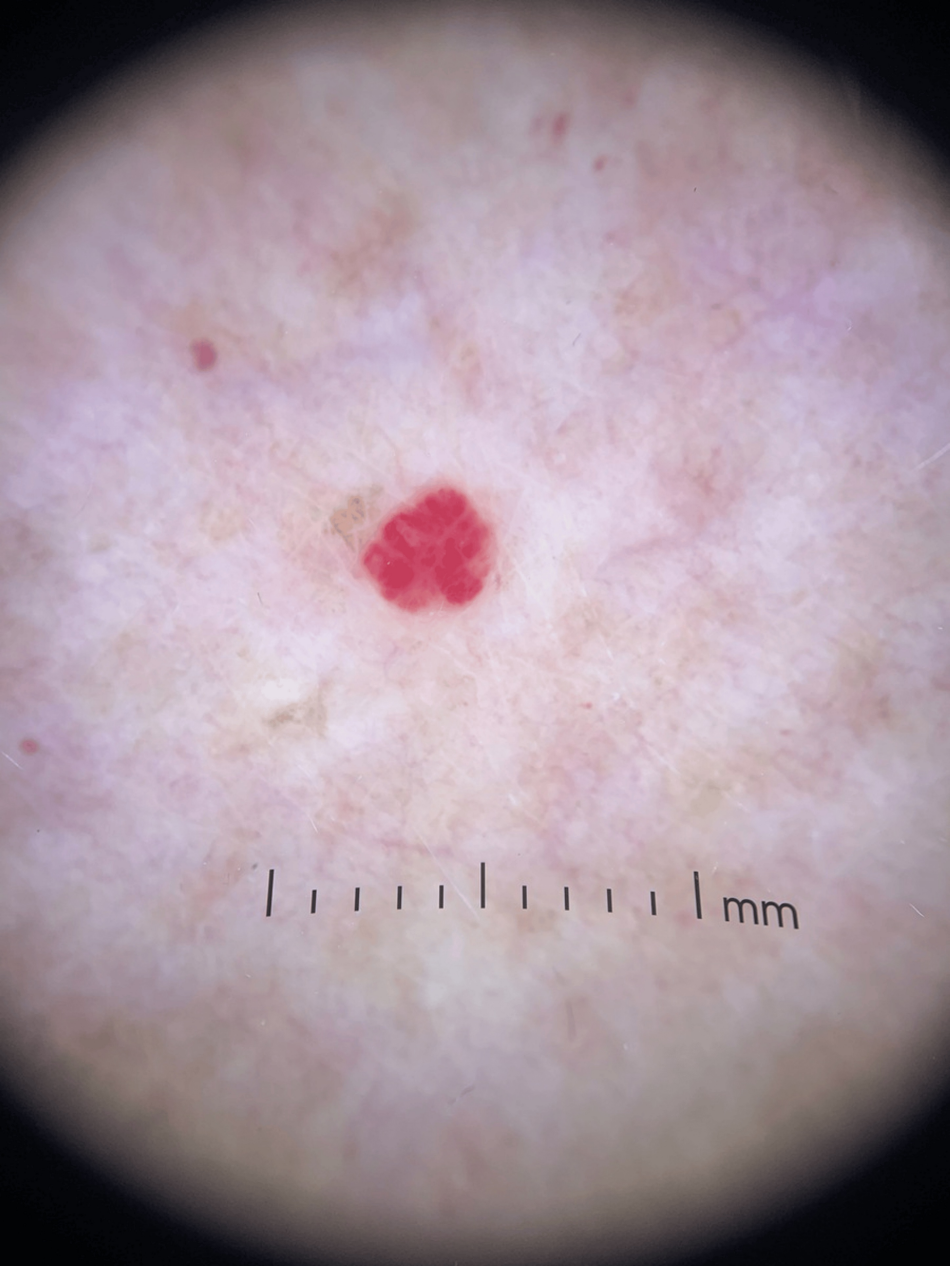
Dermoscopic view of one lesion, confirmed diagnosis of cherry hemangioma, in Patient B

Prior to this eruption, she denied viral prodrome or any history of vascular malformation. Biopsy was deferred, given the distinct banal appearance of lesions, consistent with cherry hemangiomas (Figure 3). According to the patient, the number of lesions remained stable since their initial eruption; however, they were cosmetically distressing to the patient, who requested intervention. The patient was treated with PDL using purpuric settings and has not yet followed up in clinic to assess the durability of treatment.

Patient C

A 42-year-old G4P4 female patient presented to the dermatology clinic for evaluation of suspicious nevi. She had a history of basal cell carcinoma and several severely atypical nevi, treated with wide local excision. She had no personal or family history of melanoma. She requested evaluation of an eruption of red asymptomatic papules on her trunk, which began during the third trimester of her third pregnancy at age 32. After the initial eruption, the number of lesions stabilized and did not self-resolve. During her fourth pregnancy, she suffered an acute increase in the quantity of lesions, reporting at least a doubling in number.

**Figure 4:**
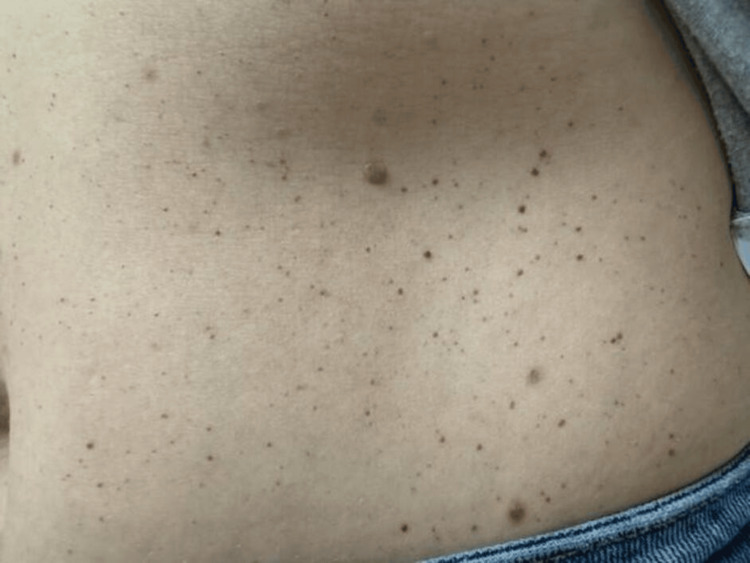
Presentation of numerous cherry hemangiomas on lateral aspect of anterior trunk in Patient C

Physical exam revealed thousands of dome-shaped papules in varying shades of red (Figure 4). The lesions predominantly affected the trunk, with decreasing density on the extremities, and were nearly absent on her head and neck. When asked about her pregnancies, she stated that the most notable difference between pregnancy three and four was that she suffered from increased nausea that extended throughout the pregnancy, which was new for her. Due to the benign presentation, no lesions were biopsied, and no further workup was performed. The lesions were cosmetically concerning to the patient, but treatment was not covered by her insurance, and the out-of-pocket cost was prohibitive. 

## Discussion

Cherry angiomas are common benign acquired vascular proliferations frequently encountered in the dermatology exam room. These bright red, dome-shaped papules are easily diagnosed clinically [4]. Some may present with a halo; however, the halo quality may not be typical for cherry angiomas [5]. Fettahlioglu Karaman, in their study on 488 patients with cherry angiomas, noted a potential positive association between halo formation around cherry angiomas and age (older than 59 years old), size (three mm), number (more than four lesions) and argued that halo formation may be a natural progression of cherry angioma evolution versus involution (such as with congenital angiomas) [4].

A common differential diagnosis, eruptive pseudoangiomatosis, is a rare, benign, short-term exanthem of angioma-like lesions that self-resolves in a matter of days to weeks [2,6]. Typically, this has been reported more often in children who present with a viral prodrome, associated with viruses such as Epstein-Barr virus, cytomegalovirus, or parvovirus, versus more of an asymptomatic state in adults. Though it has a similar clinical appearance, which may be due to direct viral interaction of endothelial cells or antigen-antibody interactions at the vascular level, histological findings reveal a lack of vascular proliferation, distinguishing it from eCA [3]. 

The term "eruptive cherry angiomatosis" refers to the sudden development of multiple cherry angiomas. However, no specific time or number of cherry angiomas has been defined for this rare condition. The underlying pathophysiology is unknown, but eCA has been associated with medications, lymphoproliferative disease, graft versus host disease, immunosuppression, and human herpesvirus-8 [4,7]. This is suggestive that a predisposing factor for eCA may be an alteration in skin immune competence in a genetically predisposed individual [7,8].

A cross-sectional study of 1302 patients by Borghi et al. in 2016 arbitrarily defined eCA as >30 CA to develop after puberty [3]. Of the 582 cases of eCA, they identified 167 with cutaneous malignancy, but did not subcategorize further based on type. Their work was expanded upon by Corazza et al., who specifically looked at the association of skin melanoma and eCA [2]. They found melanoma in 22.5% of cases of eCA as well as non-melanoma skin cancer (NMSC) in 22.8% of cases, both of which were statistically significant. Notably, the definition of eCA was modified to >10 CA to develop after puberty in their study. A study by Paolino et al. showed an association of uveal melanoma with eCA and demonstrated that eCA had a higher odds ratio of melanoma than other commonly utilized risk stratification exam findings, and that the median age of patients who had eCA with uveal melanoma was lower than that of those without [9].

The exact underlying pathophysiology for each of our patient presentations remains unclear (Table 1). Two of our patients initially presented with eCA during or right after pregnancy, while the remaining patient was possibly undergoing perimenopause. Thus, there could be a potential link between eCA and female hormones or an immunosuppression state during pregnancy. Thus, this case series suggests that female sex and hormonal changes may be underlying factors, representing a new etiology of eCA. Estrogen level rises in pregnancy as well as during the initial-mid stages of perimenopause before decreasing prior to the transition to menopause. It is this steep decrease in 17β-estradiol, the main circulating form of estrogen, that is associated with increased risk of cardiovascular disease in aging women [10].

**Table 1:** Details of eruptive cherry angiomatosis cases reported in this case series

Age, Sex	Clinical presentation	Timeframe of symptoms	Histologic result (if applicable)	Complementary tests (if applicable)	Treatment and evolution
52, F	Red papules of the bilateral upper extremity and too numerous to count 1-2 mm capillary malformations with scattered larger lesions of the trunk.	Rapid eruption over months affecting trunk and extremities in the setting of menopause	Hemangioma	Colonoscopy, endometrial biopsy, UA, genetic testing, labwork (including coagulation studies), CT abdomen/pelvis	Upper extremities and trunk treated with PDL x3. Lesions recur in treated areas. Stable number of lesions
38, F	Multiple small dome shaped, symmetric red papules with well demarcated borders of the trunk.	Lesions noted after delivery of first child in July 2022.	NA	None	Lesions persistent; initial PDL treatment performed at last office visit.
32, F	Multiple polymorphic red papules on the trunk and the extremities	Initial acute onset during the third trimester of 3rd pregnancy. Second eruption during the fourth pregnancy.	NA	None	Lesions have remained stable after delivery of the 4th child. No interventions.

Estrogen is a hormone with a great influence on the body, for which its effects on angiogenesis are still being theorized. There are three types of estrogen receptors: estrogen receptor alpha, estrogen receptor beta, and G protein-coupled estrogen receptor 1. All three receptors promote vascular health through effects such as reducing calcification and endothelial dysfunction, while stimulating vasodilation and vascular endothelial growth factor [11]. Because of these vascular effects, we theorize that increased estrogen promoted angioma growth in our patients. 

## Conclusions

Though limited by its observational nature and small sample size, this report highlights an important distinction with eCA, which should guide clinical decision-making and counseling. A thorough history and workup should be obtained, as well as awareness that a pale halo is more likely associated with pseudoangiomatosis than cherry angiomas. Early recognition and counseling are important in diagnosing eCA vs other vascular exanthems such as pseudoangiomatosis. 

As noted with our patients, eCA lesions will likely persist for months to years and recur despite treatment. Given their chronic yet benign nature, reassurance and discussion early on with patients will help reduce patient distress, unnecessary follow-ups, and waste of resources. As part of our patient counseling, given the associated increased risk of malignant melanoma, uveal melanoma, and non-melanoma skin cancer, our patients were educated accordingly on this as well.
